# Effect of a spore-based *Mannheimia haemolytica* vaccine on immune responses and respiratory microbiota in sheep

**DOI:** 10.1038/s41541-026-01415-x

**Published:** 2026-07-10

**Authors:** Muhammed Salah Uddin, José Ortiz Guluarte, Timothy D. Schwinghamer, Daniel R. Barreda, Tim A. McAllister, Le Luo Guan, Trevor W. Alexander

**Affiliations:** 1 Lethbridge Research and Development Centre, Agriculture and Agri-Food Canada, Lethbridge, AB Canada; 2 Department of Agricultural, Food and Nutritional Science, University of Alberta, Edmonton, AB Canada; 3 Department of Biological Sciences, University of Alberta, Edmonton, AB Canada; 4 Faculty of Land and Food Systems, University of British Columbia, Vancouver, BC Canada

**Keywords:** Diseases, Immunology, Microbiology

## Abstract

*Mannheimia haemolytica* is an opportunistic pathogen associated with respiratory disease in ruminants. Current vaccines provide incomplete protection, highlighting the need for improved immunization strategies. In this study, a mucosal vaccine was developed using *Bacillus subtilis* spores as an adjuvant and evaluated for its effects on immune responses and respiratory microbiota in sheep. A chimeric protein (MhCP) containing neutralizing epitopes from leukotoxin A (NLKT) and outer membrane protein PlpE was expressed and adsorbed onto spores to produce Spore-MhCP, which was administered via two mucosal routes: intranasal and intragastric. Unbound MhCP was delivered intranasally and intramuscularly, while unbound spores and saline were used as controls. Intranasal Spore-MhCP generated the strongest secretory IgA-specific responses against PlpE and NLKT in nasal swab, bronchoalveolar lavage, and fecal samples. It also elicited earlier and sustained serum IgG responses among mucosal immunization groups. Notably, intragastric vaccination also increased PlpE- and NLKT-specific antibodies in lung and fecal samples. From nasopharyngeal samples, 16S rRNA gene sequencing revealed 27 genera altered in the intranasal Spore-MhCP group, including a decrease in *Mannheimia* (days 14–35; *p* < 0.01). These findings indicate that intranasal Spore-MhCP enhances immunity and may reduce *M. haemolytica* proliferation in the upper respiratory tract, reducing lung infection risk.

## Introduction

*Mannheimia haemolytica* is a predominant bacterial pathogen associated with severe pneumonia in ruminants, affecting cattle, goats, and sheep^1–4^. This bacterium is a primary opportunistic pathogen in bovine respiratory disease (BRD), a significant contributor to cattle morbidity and mortality^5^, and also causes mastitis in ewes and septicemia in lambs^2,4^. While various serotypes of *M. haemolytica*, including A1 and A2, can be found in the upper respiratory tract of healthy ruminants, serotypes A1 and A6 are mainly responsible for bovine bronchopneumonia, whereas serotypes A1 and A2 are primarily associated with pneumonia in sheep^2,5,6^. Current commercial *M. haemolytica* vaccines have exhibited variable performance in field trials measuring pneumonia in cattle^7–14^, highlighting the need for novel vaccines with improved efficacy. An ideal vaccine would be capable of eliciting both mucosal and systemic immune responses, providing localized protection at the infection site in addition to systemic immunity.

*M. haemolytica* virulence factors, including leukotoxin (LKT), outer membrane proteins, lipopolysaccharides, iron-binding proteins, and capsular polysaccharides, are key for pathogenesis and are prime targets of the host immune response, making them potential antigen candidates^2,15,16^. Several in-vivo studies with LKT-deletion mutants have identified LKT as the dominant virulence factor^3,17,18^, highlighting its significance in pathogenesis and importance as a preferred antigen for vaccine development^2^. Additionally, several outer membrane lipoproteins have been reported as immunologically key surface antigens^19–22^. We previously developed a spore-based mucosal vaccine designated as “Spore-MhCP” against *M. haemolytica* and demonstrated its ability to stimulate both systemic (IgG) and secretory (IgA) antibody responses following mucosal immunization in a mouse model^23^. This vaccine was prepared by purifying a chimeric protein, comprising repeated neutralizing antigenic epitopes from *M. haemolytica* leukotoxin A (NLKT) and outer membrane protein PlpE^2,23^, which was subsequently adsorbed to *Bacillus subtilis* spores. The NLKT and PlpE antigens in Spore-MhCP were designed to be conserved across *M. haemolytica* strains pathogenic to bovine, mainly serotypes 1 and 6^2,20,23^. Effective delivery systems are essential for maintaining the antigenicity of chimeric protein vaccines^24^. *Bacillus subtilis* spores, known for their ability to interact with host mucosa and survive in diverse environmental conditions^25,26^, have been investigated as a vaccine vehicle. Several studies have demonstrated that mucosal immunization with *B. subtilis* spores coated with antigens can elicit strong antibody responses, thereby conferring protection against various infections^26–29^. Although the goal is for Spore-MhCP to be used in cattle, in this study, the vaccine was further evaluated in sheep for immunogenicity when administered by different delivery routes. Sheep were used as a cattle model due to their natural colonization by *M. haemolytica*, their similarities in rumination, and their use as a more cost-effective model compared to cattle.

Interactions between host microbiota and immune cells can impact vaccine effectiveness^30,31^. However, the direct or indirect effects of vaccination on respiratory tract microbiota are still underexplored. Therefore, we evaluated the upper respiratory microbiota from sheep nasopharyngeal samples to assess the impact of this mucosal vaccine on bacterial microbiota. Based on the evidence that intranasal administration of Spore-MhCP induced both mucosal and systemic immune responses in mice, we hypothesized that immunization with intranasally delivered Spore-MhCP would elicit similar immune responses in sheep and effectively reduce the relative abundance of *Mannheimia* in the upper respiratory tract. Therefore, the objectives of this study were to firstly, evaluate the immunogenicity of Spore-MhCP vaccine in sheep after delivery through oral or intranasal routes and secondly, explore the effect of Spore-MhCP vaccine on the sheep respiratory microbiota.

## Results

### Antigen-specific antibody production in serum

**Fig. 1:**
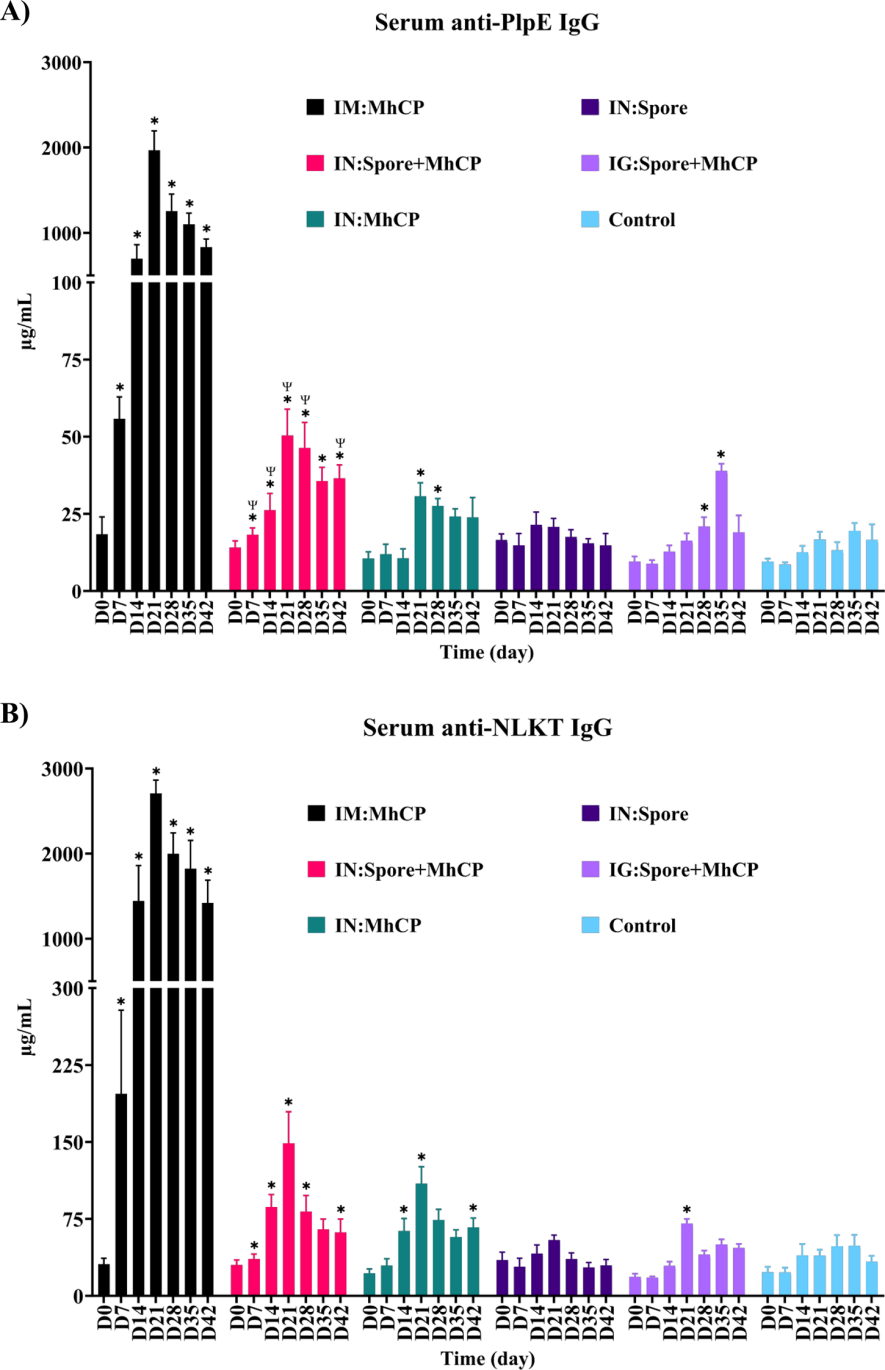
Antigen-specific serum IgG responses in sheep. *Mannheimia haemolytica* chimeric protein MhCP (CTB+PlpE+NLKT+PlpE+NLKT) was used to immunize sheep via different routes either in free form or after bound to *B. subtilis* spores. A total of 48 sheep were divided into six experimental groups (N = 8 sheep per treatment): Intramuscular free antigen (IM:MhCP), Intranasal spore-bound antigen (IN:Spore+MhCP), Intranasal free antigen only (IN:MhCP), Intranasal spore only (IN:Spore), Intragastric spore-bound antigen delivered in feed (IG:Spore+MhCP) and Control/Naïve sheep (Control; no antigen used, negative control). Sheep were immunized on days 0 and 14, with the exception of the IG:Spore+MhCP (in-feed) group, which was vaccinated on days 1, 2, 14, 15, 28 and 29. Blood samples were collected on days 0, 7, 14, 21, 28, 35, and 42, and sera was isolated and analyzed by enzyme-linked immunosorbent assay (ELISA) to measure anti-PlpE and anti-NLKT antibodies. IgG specific to PlpE (A) and NLKT (B) are shown (mean ± SEM). * *p* < 0.05 when compared to the same timepoint of the control treatment. ^Ψ^ *p* < 0.05 when compared between two intranasal treatment groups (IN:Spore+MhCP vs IN:MhCP).

On day 0, all sheep had low but detectable concentrations of serum IgG against *M. haemolytica* antigens NLKT and PlpE (Fig. 1). All the treatments carrying antigen MhCP had higher concentrations of serum anti-PlpE and anti-NLKT IgG on at least one timepoint, compared to the Control treatment (Fig. 1). No significant differences in serum IgG concentrations were observed between the Control and IN:Spore groups at any assessed time point (*p* > 0.05). Intramuscular immunization with MhCP on day 0, resulted in increased serum antibody concentrations in the IM:MhCP group against both NLKT and PlpE by day 7 (*p* < 0.05; Fig. 1). A booster dose on day 14 further enhanced immune responses in the IM:MhCP treatment group, such that maximum antibody concentrations were detected on day 21 against both NLKT and PlpE. The immune responses in the IM:MhCP group remained significantly higher until the end of the study period (day 42), as compared to all other treatment groups (*p* < 0.05). The IN:Spore+MhCP group reached maximum IgG antibody concentrations on day 21 against PlpE and NLKT after receiving the booster dose on day 14, and remained higher (*p* < 0.05) until study end as compared to the Control treatment. After receiving the booster immunization, the IN:MhCP treatment group also showed higher anti-PlpE and anti-NLKT IgG antibody responses on day 21 (*p* < 0.05), compared to the Control treatment. When comparing these two antigen-carrying intranasal treatment groups, IN:Spore+MhCP induced higher anti-PlpE IgG concentrations (*p* < 0.05) at most evaluated time points. Notably, intragastric (in-feed) vaccination resulted an increased anti-PlpE IgG concentrations on days 28 and 35 (*p* < 0.05), when compared to the Control treatment. Overall, among the treatment groups immunized via oral or intranasal routes, the IN:Spore+MhCP group exhibited a tendency to increase serum IgG concentrations more rapidly than other groups and maintained higher levels compared to the Control treatment.

### MhCP induced production of antigen‑specific secretory antibodies

**Fig. 2:**
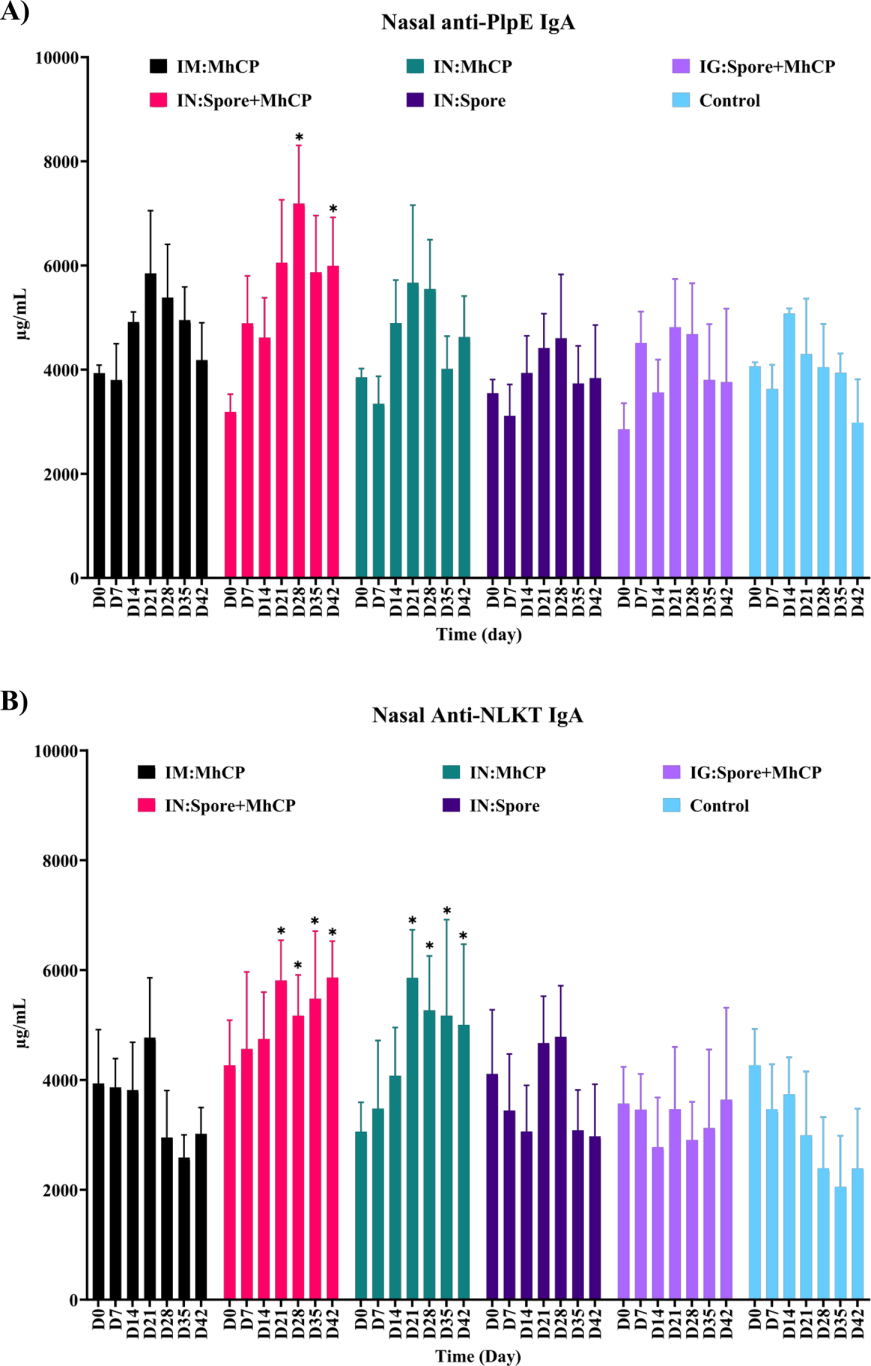
Mucosal IgA immune responses from sheep nasal secretions. *Mannheimia haemolytica* chimeric protein MhCP (CTB+PlpE+NLKT+PlpE+NLKT) was used to immunize sheep via different routes either in free form or after bound to *B. subtilis* spores. A total of 48 sheep were divided into six experimental groups (*N* = 8 sheep per treatment): Intramuscular free antigen (IM:MhCP), Intranasal spore-bound antigen (IN:Spore+MhCP), Intranasal free antigen only (IN:MhCP), Intranasal spore only (IN:Spore), Intragastric spore-bound antigen delivered in feed (IG:Spore+MhCP) and Control/Naïve sheep (Control; no antigen used, negative control). Sheep were immunized on days 0 and 14, with the exception of the IG:Spore+MhCP (in-feed) group, which was vaccinated on days 1, 2, 14, 15, 28 and 29. Nasal samples were collected on days 0, 7, 14, 21, 28, 35, and 42 and analyzed by ELISA to measure anti-PlpE and anti-NLKT antibodies. IgA specific to PlpE (A) and NLKT (B) are shown (mean ± SEM). *p < 0.05 when compared to the same timepoint of the control treatment.

In nasal samples, all the treatment groups showed detectable concentrations of PlpE- and NLKT-specific IgA on day 0, indicating that sheep in this study had an immune response to *M. haemolytica* colonization prior to study initiation (Fig. 2). Both of the spore-bound groups, IN:Spore+MhCP and IG:Spore+MhCP, exhibited a significant increase in anti-PlpE IgA concentrations over time compared to their pre-immunization levels (Fig. 2A). Compared to day 0, IN:Spore+MhCP group exhibited increased concentrations across all the time points (*p* < 0.05), and the IG:Spore+MhCP group showed higher anti-PlpE IgA on days 7, 21 and 28 (*p* < 0.05). IM:MhCP and IN:MhCP groups also showed a limited increase of anti-PlpE IgA concentrations (*p* > 0.05), after receiving the booster immunization. However, unlike the IN:Spore+MhCP group, the antibody concentrations of these groups did not remain consistent until study completion and almost returned to pre-booster levels over time. No other treatment groups showed a significant increase in their anti-PlpE IgA concentrations compared to their initial levels. After receiving the initial immunization on day 0 and booster dose on day 14, the IN:Spore+MhCP group reached the highest anti-PlpE IgA concentrations on day 28, and it was the only group that showed higher anti-PlpE IgA at two time points when compared to Control treatment (*p* < 0.05; Fig. 2A). After receiving the booster immunization, both the IN:Spore+MhCP and IN:MhCP groups exhibited higher anti-NLKT IgA on days 21 to 42, when compared to the Control group (*p* < 0.05; Fig. 2B). No other treatment groups demonstrated a significant increase in their anti-NLKT IgA concentrations compared to the control treatments or their initial levels. Overall, the IN:Spore+MhCP group exhibited a tendency to enhance the immune response faster than other treatments and maintained a higher PlpE- and NLKT-specific IgA until study end compared to the Control treatment.

**Fig. 3:**
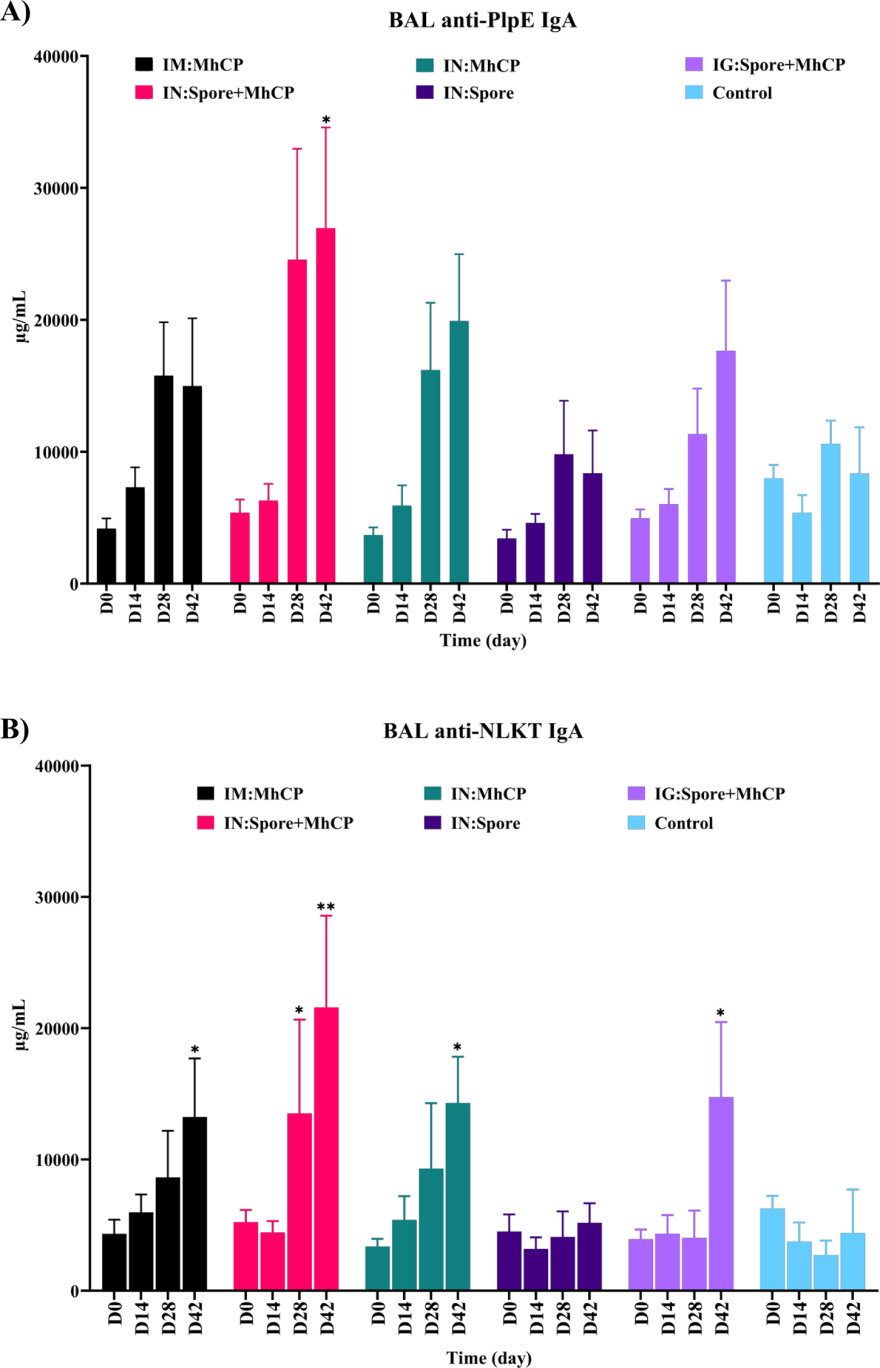
Mucosal IgA immune responses from sheep Bronchoalveolar lavage (BAL). *Mannheimia haemolytica* chimeric protein MhCP (CTB+PlpE+NLKT+PlpE+NLKT) was immunized via different routes, either in free form or after bound to *B. subtilis* spores. A total of 48 sheep were divided into six experimental groups (*N* = 8 sheep per treatment): Intramuscular free antigen (IM:MhCP), Intranasal spore-bound antigen (IN:Spore+MhCP), Intranasal free antigen only (IN:MhCP), Intranasal spore only (IN:Spore), Intragastric spore-bound antigen delivered in feed (IG:Spore+MhCP) and Control/Naïve sheep (Control; no antigen used, negative control). Sheep were immunized on days 0 and 14, with the exception of the IG:Spore+MhCP (in-feed) group, which was vaccinated on days 1, 2, 14, 15, 28 and 29. BAL samples were collected on days 0, 14, 28, and 42 and analyzed by ELISA to measure anti-PlpE and anti-NLKT antibodies. IgA specific to PlpE (**A**) and NLKT (**B**) are shown (mean ± SEM). * *p* < 0.05, ** *p* < 0.01 when compared to the same timepoint of Control treatment.

Similar to nasal samples, sheep from all treatment groups had quantifiable PlpE- and NLKT-specific IgA in BAL samples on day 0, indicative of an immune response to *M. haemolytica* before study initiation (Fig. 3). With the exception of the Control group, all the other treatment groups showed an increased anti-PlpE IgA concentration on days 28-42, as compared to their pre-immunization levels (*p* < 0.05; Fig. 3A). However, IN:Spore+MhCP was the only treatment group that showed higher anti-PlpE IgA on day 42, when compared to Control treatment (*p* < 0.05). None of the other treatment groups showed increased anti-PlpE IgA concentration compared to the control treatment at any time points (*p* > 0.05; Fig. 3A). Similar trends were observed while measuring the NLKT-specific IgA from BAL samples (Fig. 3B). All the antigen MhCP carrying groups showed an increased anti-NLKT IgA concentration on day 42, when compared to their pre-immunization levels (*p* < 0.05; Fig. 3B). IN:Spore and Control treatment groups did not increase anti-NLKT IgA concentration compared to their levels on day 0. Apart from the IN:Spore treatment group, all the other groups carrying MhCP showed a greater anti-NLKT IgA level on day 42 compared to the Control group (*p* < 0.05; Fig. 3B). However, immune responses in the IN:Spore+MhCP group were greatest both on days 28 and 42, indicating an earlier immune response in this group. Notably, the IG:Spore+MhCP group showed a sharp spike of anti-NLKT IgA on day 42, as compared to the Control treatment or to the earlier time points for this group (*p* < 0.05).

**Fig. 4:**
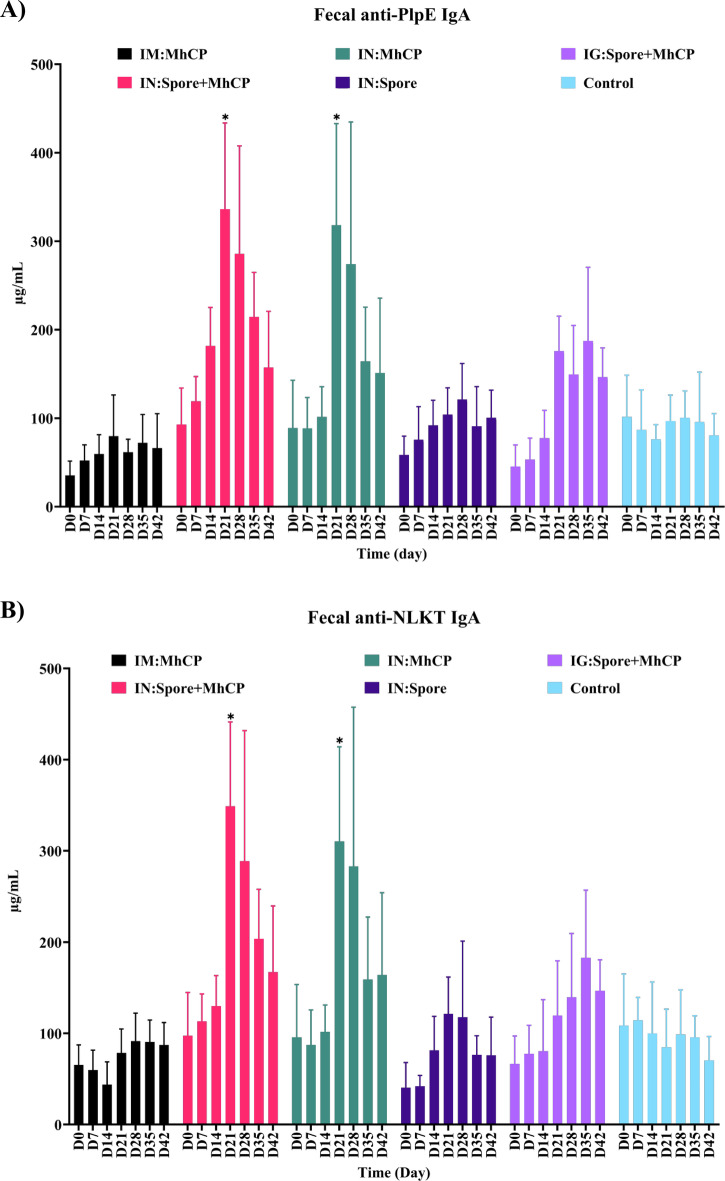
Mucosal IgA immune responses from sheep feces. *Mannheimia haemolytica* chimeric protein MhCP (CTB+PlpE+NLKT+PlpE+NLKT) were immunized via different routes either in free form or after bound to *B. subtilis* spores. A total of 48 sheep were divided into six experimental groups (*N* = 8 sheep per treatment): Intramuscular free antigen (IM:MhCP), Intranasal spore-bound antigen (IN:Spore+MhCP), Intranasal free antigen only (IN:MhCP), Intranasal spore only (IN:Spore), Intragastric spore-bound antigen delivered in feed (IG:Spore+MhCP) and Control/Naïve sheep (Control; no antigen used, negative control). Sheep were immunized on days 0 and 14, with the exception of the IG:Spore+MhCP (in-feed) group, which was vaccinated on days 1, 2, 14, 15, 28 and 29. Fecal samples were collected on days 0, 7, 14, 21, 28, 35, and 42 and analyzed by ELISA to measure anti-PlpE and anti-NLKT antibodies. IgA specific to PlpE (A) and NLKT (B) are shown (mean ± SEM). * *p* < 0.05 when compared to the same timepoint of the control treatment.

In fecal samples, both IN:Spore+MhCP and IN:MhCP groups reached their maximum anti-PlpE IgA titers on day 21, after receiving the booster immunization, and remained higher until day 35 as compared to their levels on day 0 (*p* < 0.05; Fig. 4A). However, antibody concentrations for both of these groups did not remain elevated until the end of the study, although it was numerically higher compared to their initial levels (*p* > 0.05). Compared to the pre-immunization levels, IG:Spore+MhCP group showed a higher anti-PlpE IgA by day 21 (*p* < 0.05), and remained higher until end of the study (day 42). Compared to Control treatment, IN:Spore+MhCP and IN:MhCP groups showed higher anti-PlpE IgA concentrations only at one time point on day 21 (*p* < 0.05; Fig. 4A). Compared to day 0, the three intranasal treatment groups and the IG:Spore+MhCP group showed higher anti-NLKT IgA concentrations by day 21 (*p* < 0.05) and remained elevated until the end of the study (Fig. 4B). However, when compared to Control treatment, IN:Spore+MhCP and IN:MhCP groups showed higher anti-NLKT IgA concentrations on day 21 (*p* < 0.05; Fig. 4B). None of the other treatment groups demonstrated increased immune responses compared to the control treatment at any time points.

### The community structure of sheep respiratory microbiota

The upper respiratory tract microbiota of sheep was analyzed in control and intranasal Spore-MhCP groups across time, by sequencing the 16S rRNA gene of nasal samples. Pre-processing steps produced 1,901 ASVs in 84 samples from 1,912,730 merged paired reads. At this stage, the median number of sequences per sample was 21902.5 ± 7229, with a minimum of 9599 and a maximum of 41,001. After removing the ASVs present in less than 1% of the samples, 432 ASVs comprised of 1,843,341 reads remained. The final median number of sequences was 20,732 ± 7382 with a minimum of 6483 and a maximum of 40,772 sequences per sample.

**Fig. 5:**
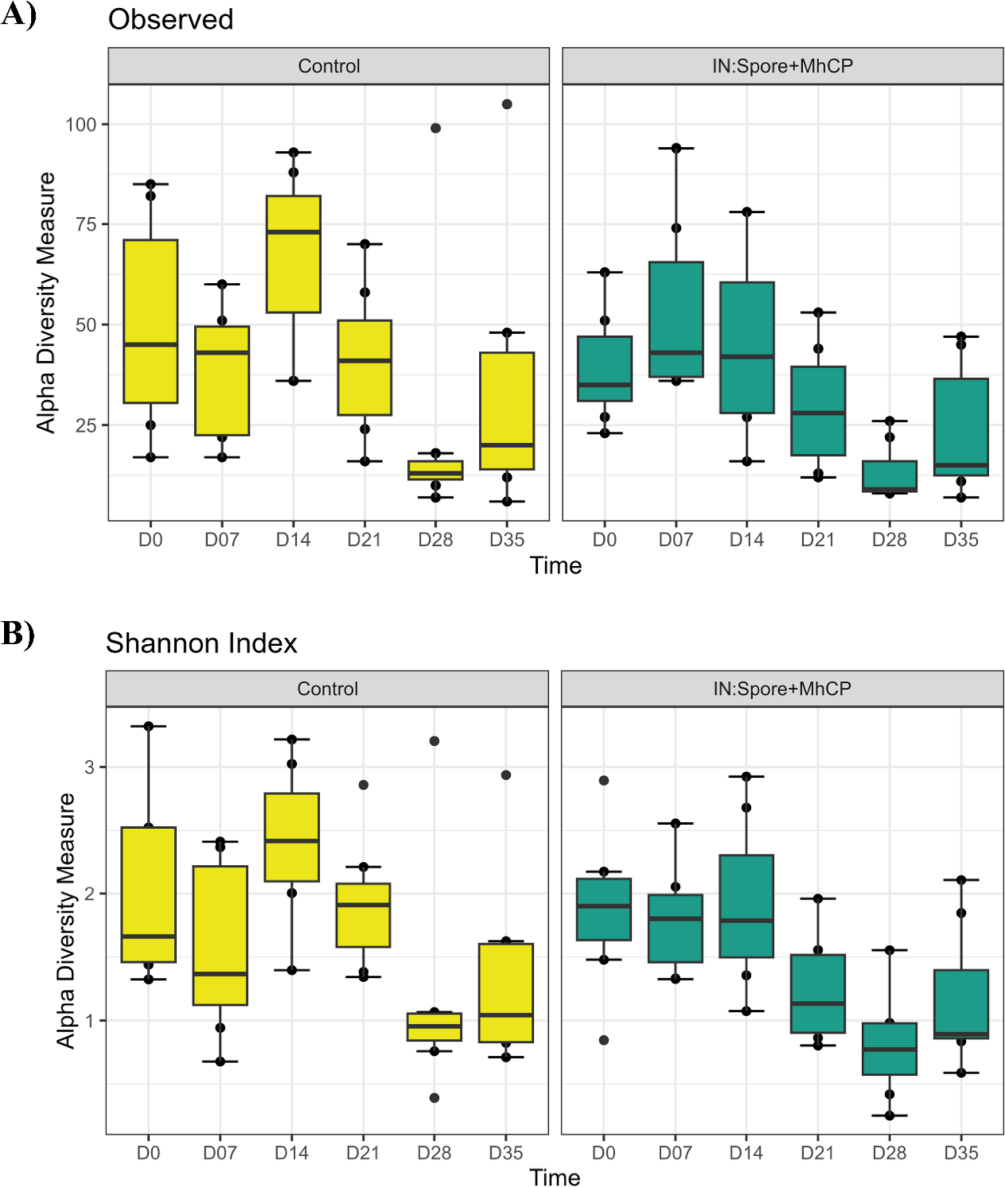
Alpha diversity of nasopharyngeal bacteria from vaccinated sheep. Upper plot shows the bacterial richness or counts of taxa observed (**A**), and lower plot shows the Shannon diversity index (**B**). The box in the plots indicates the interquartile range (IQR; middle 50% of the data), the middle line represents the median value, and the whiskers represent 1.5 times the IQR. Sheep were immunized on days 0 and 14. Deep nasal swabs (from right nostril) were collected from Intranasal spore-bound antigen (IN:Spore+MhCP) and Control/ Naïve (Control; administered intranasal saline) treatment groups on days 0, 7, 14, 21, 28, and 35 and analyzed by sequencing of the 16S rRNA gene.

ANOVA testing of alpha diversity revealed that sampling time had a significant effect on richness and Shannon index (*p* < 0.001). The diversity on day 28 significantly decreased compared to days 0, 7, and 14, whereas day 35 was significantly lower compared to day 14 (*p* < 0.05; Fig. 5). Overall, the diversity decreased across time. These differences over time were observable in the plotted richness and Shannon index metrics (Fig. 5A and 5B). IN:Spore+MhCP was also determined to significantly lower mean Shannon diversity index when compared to the control (*p* < 0.05).

**Fig. 6:**
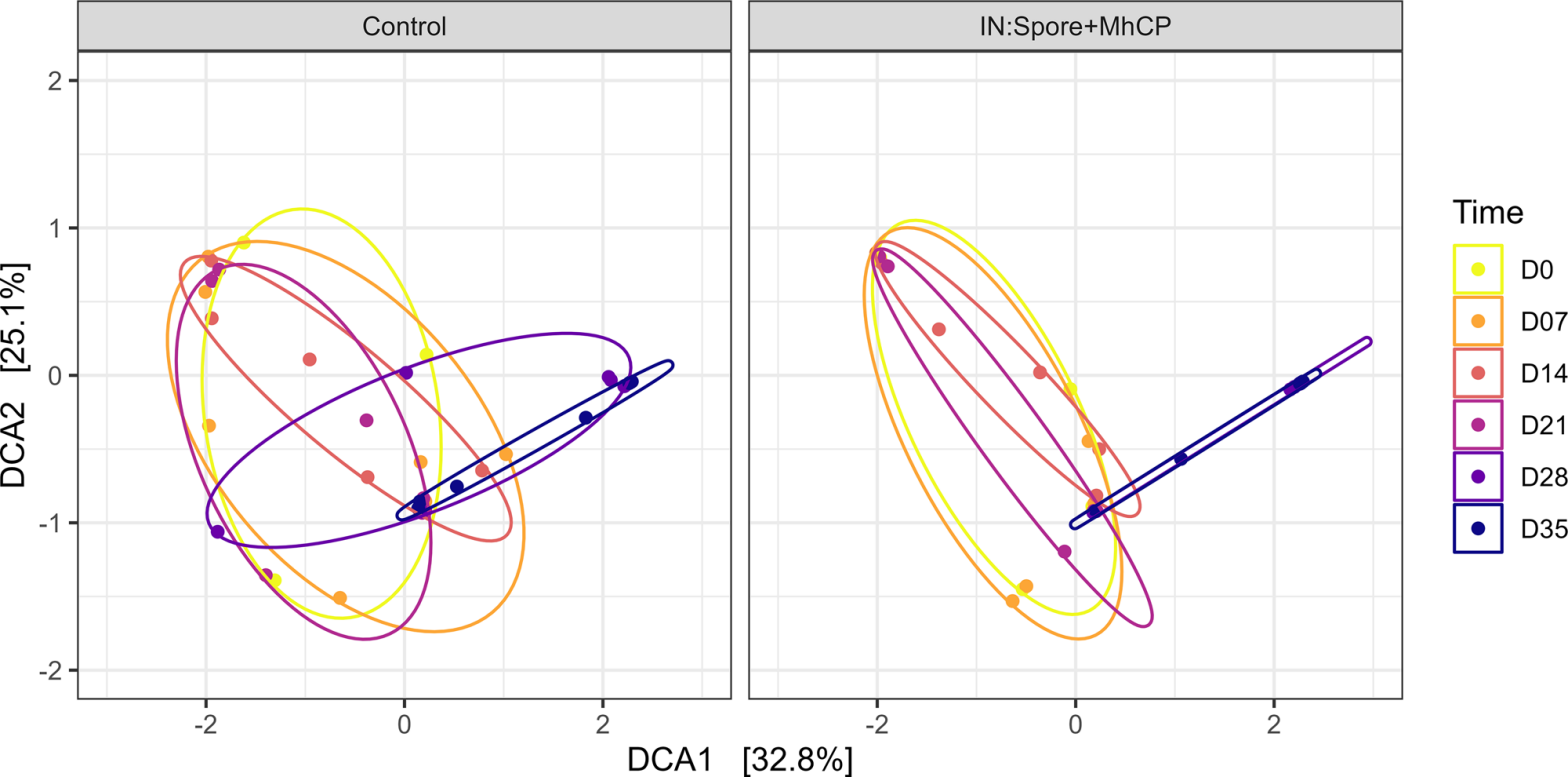
Detrended correspondence analysis (DCA) plots of the Bray-Curtis metric of nasopharyngeal samples from vaccinated sheep. Sheep were immunized on days 0 and 14. Deep nasal swabs (from right nostril) were collected from Intranasal spore-bound antigen (IN:Spore+MhCP) and Control/ Naïve (Control; administered intranasal saline) treatment groups on days 0, 7, 14, 21, 28, and 35 and analyzed by sequencing of the 16S rRNA gene.

PERMDISP testing with respect to time and treatment variables determined that only time was significant (*p* < 0.001) suggesting that sampling times do not have homogenous dispersion of microbial communities. Further testing with PERMANOVA identified that sampling time was significant (R2 = 0.361, *p* < 0.001), but treatment and the interaction of treatment and time were not. These results further verified that there were significant differences in microbial communities between sampling times. These trends were observed within the DCA plots as the samples within each treatment clustered differently with time (Fig. 6).

### Composition of sheep respiratory microbiota

**Fig. 7:**
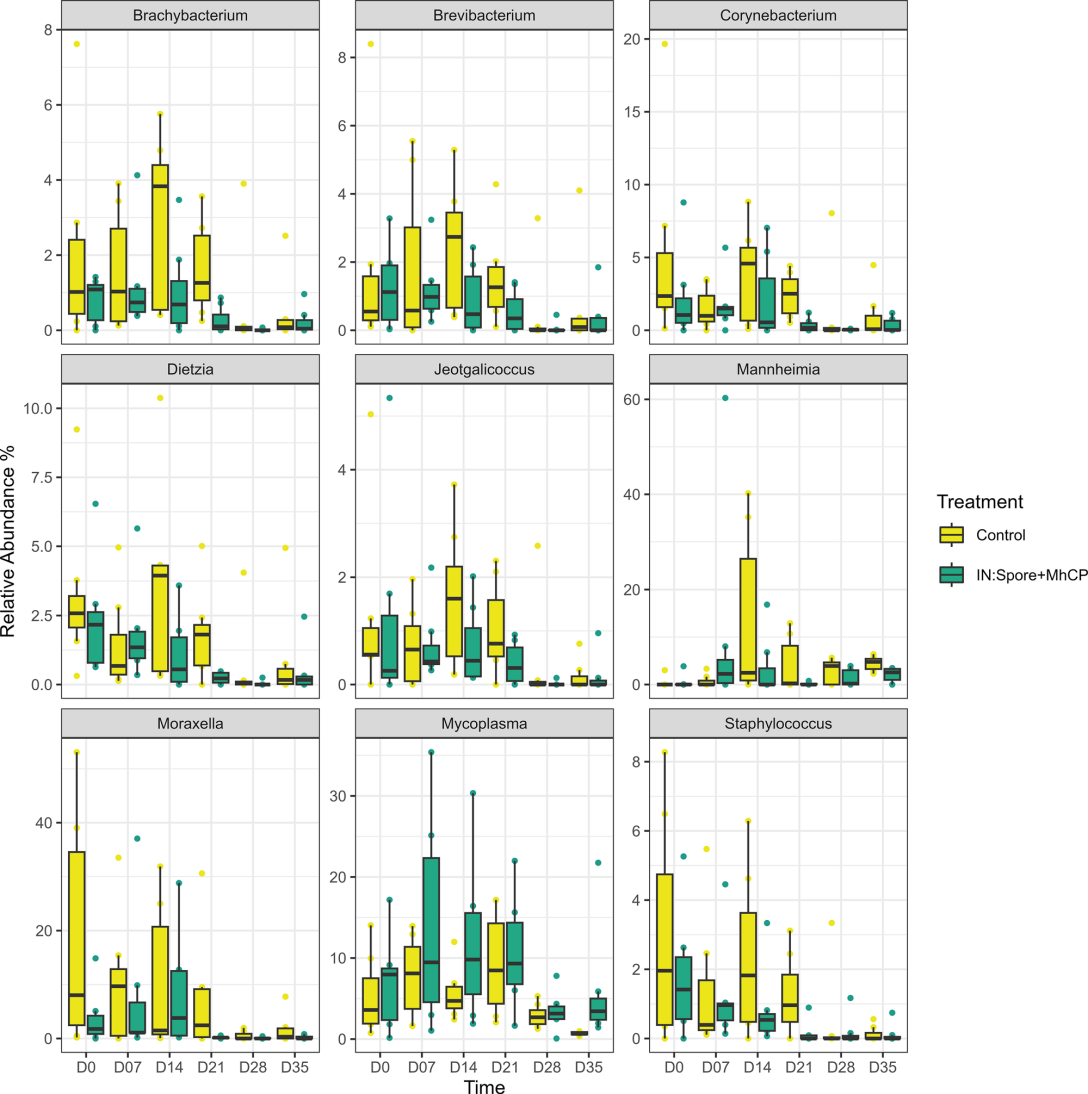
Relative abundance of the top nine most abundant genera identified in nasopharyngeal samples from vaccinated sheep. Sheep were immunized on days 0 and 14. Deep nasal swabs (from right nostril) were collected from Intranasal spore-bound antigen (IN:Spore+MhCP) and Control/ Naïve (Control; administered intranasal saline) treatment groups on days 0, 7, 14, 21, 28, and 35 and analyzed by sequencing of the 16S rRNA gene. The box in the plots indicates the interquartile range (IQR; middle 50% of the data), the middle line represents the median value, and the whiskers represent 1.5 times the IQR.

Across the treatment groups and sampling times, a total of 20 different bacterial phyla were identified, and unidentifiable reads were classified as “Unclassified”. The percentage of reads of the ten most abundant phyla were: Proteobacteria, 69.84%; Bacteroidota, 11.38%; Firmicutes, 11.05%; Actinobacteriota, 6.63%; Euryarchaeota, 0.12%; Planctomycetota, 0.05%; Chloroflexi, 0.04%; Spirochaetota, 0.01%; Deinococcota, 0.01%; Verrucomicrobiota, 0.01%; and Unclassified, 0.83%. The relative abundance of Proteobacteria increased in both treatments at days D28 and D35 compared to their initial time points (Supplementary Fig. 1). When evaluated across sampling time and treatment groups, a total of 195 genera were identified. The top 9 most abundant classified genera and their relative abundance in the dataset were: *Mycoplasma*, 7.03%; *Moraxella*, 5.17%; *Mannheimia*, 3.89%; *Corynebacterium*, 1.62%; *Dietzia*, 1.34%; *Brevibacterium*, 1.03%; *Brachybacterium*, 0.98%; *Staphylococcus*, 0.94%; and *Jeotgalicoccus*, 0.61% (Fig. 7).

### Changes in sheep respiratory microbiota across sampling time and within the treatments

**Fig. 8:**
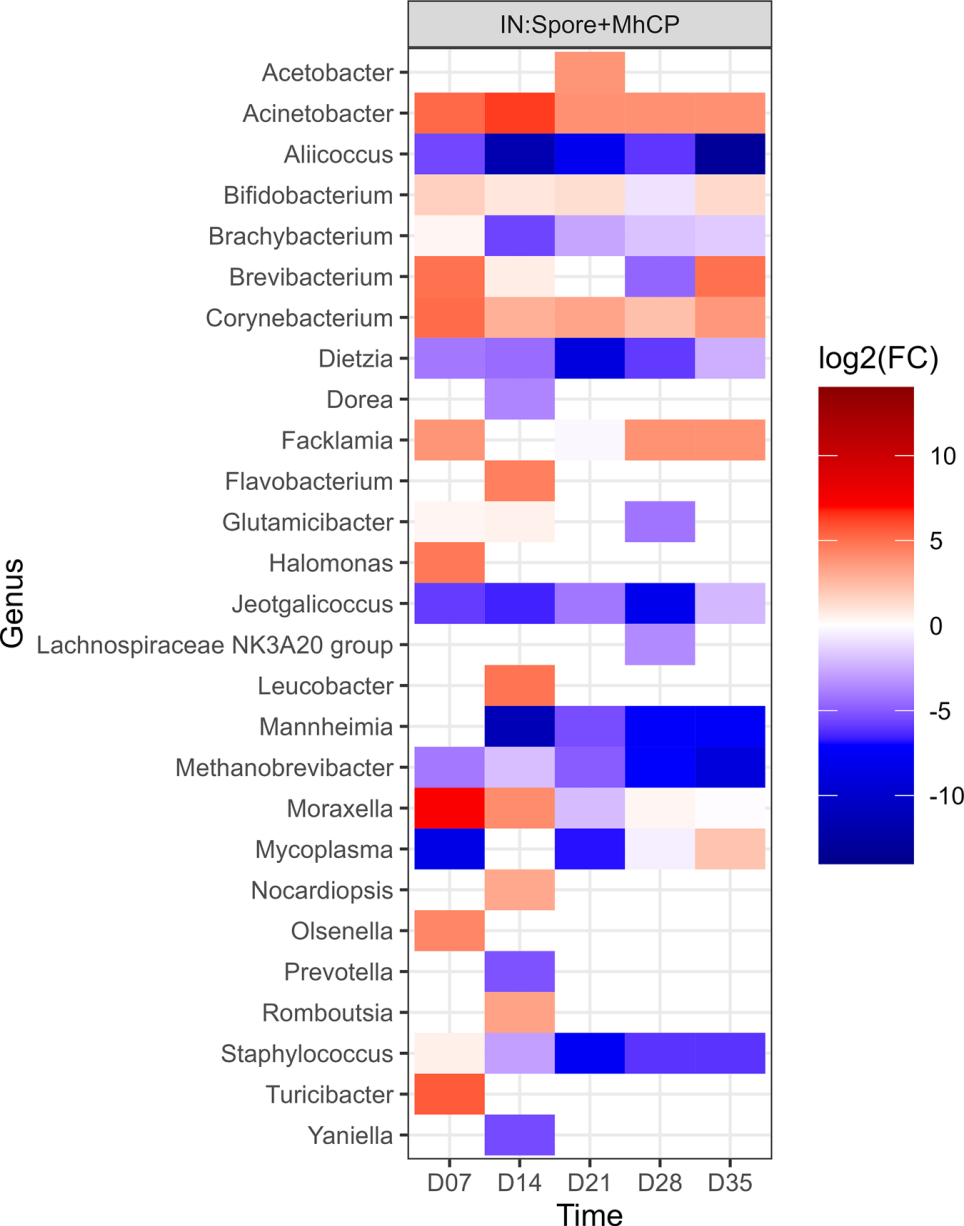
Genera that showed a significant change (*P* < 0.01, log2(FC) > 2 or log2(FC) < -2) from baseline (day 0) in IN:Spore+MhCP treatment group above and beyond any changes in the Control sheep over the course of 35 days. The heatmap shows changes in genera when microbiota of the IN:Spore+MhCP group were compared to the Control group, after both treatments were normalized to time point day 0. Sheep were immunized on days 0 and 14. Deep nasal swabs (from right nostril) were collected from Intranasal spore-bound antigen (IN:Spore+MhCP) and Control/ Naïve (Control; administered intranasal saline) treatment groups on days 0, 7, 14, 21, 28, and 35 and analyzed by sequencing of the 16S rRNA gene.

A total of 27 genera were identified that showed a significant change (*p* < 0.01, log2(FC) > 2 or log2(FC) < -2) from baseline (day 0) in the IN:Spore+MhCP treatment above and beyond any changes in the Control sheep over the course of 35 days (Fig. 8). For the IN:Spore+MhCP group, 7 genera increased at more than a single time point, while 8 genera decreased for more than one time point and almost all of them remained lower until day 35. Notably, *Mannheimia* were consistently decreased over time in IN:Spore+MhCP group, compared to Control. The relative abundance of *Aliicoccus*, *Brachybacterium, Dietzia, Jeotgalicoccus, Methanobrevibacter, Mycoplasma, and Staphylococcus* also decreased at most time points. In contrast, *Acinetobacter*, *Bifidobacterium*, *Brevibacterium*, *Corynebacterium*, and *Facklamia* increased at most time points.

## Discussion

In this study, immunization of sheep with both unbound MhCP and Spore-MhCP resulted in the production of specific antibodies against NLKT and PlpE, indicating that the chimeric antigen MhCP maintained its immunogenicity even after being adsorbed onto spores, and that the antigen constructs individually induced seroconversion. Chimeric protein possess versatility, facilitating the delivery of multiple antigens to the immune system, rendering them promising candidates for vaccine development^32,33^. Previous studies established that components of the chimeric protein MhCP, i.e., NLKT and PlpE, were immunogenic^23,34,35^, and conserved across multiple *M. haemolytica* serotypes, particularly serotypes 1 and 6, which are most often linked to morbidity and mortality in BRD^2,20^. This study further validates this finding, as evidenced by the induction of immune responses against NLKT and PlpE in sheep following intramuscular immunization with unbound MhCP. Sheep are naturally colonized by *M. haemolytica*, with serotype 2 being commonly pathogenic in this species. In this study, both PlpE- and NLKT-specific IgA were observed on day 0 (before first immunization), in all mucosal samples across all treatment groups, suggesting pre-existing immune responses to *M. haemolytica* colonization before the study commenced. This was supported by sequencing analysis of the nasal samples from IN:Spore+MhCP and Control groups, where *Mannheimia* was evident on day 0. Although the initial immunization on day 0 did not induce a significant mucosal immune response in treatment groups, the booster dose elicited a sharp spike of the antibody concentrations. The delayed immune response observed during the initial two weeks could be attributed to the heightened levels of early IgA specific to *M. haemolytica* antigens. This could have potentially hindered the mucosal antibody responses following the first immunization. The finding of an initial delayed immune response in the current study aligns with previous research findings^35,36^.

All treatments containing the MhCP antigen resulted in increased systemic immune response. The IN:Spore+MhCP group showed the earliest increase in specific IgG on day 7 among mucosal routes, when compared to Control, indicating a rapid immune response. In addition, intranasal administration of Spore-MhCP induced greater PlpE-specific IgG compared to unbound MhCP, though this was not the case for NLKT-specific IgG. The IN:Spore-MhCP group resulted in stronger secretory IgA immune responses across all evaluated mucosal samples, including nasal, BAL and fecal samples, demonstrating robust mucosal and systemic immune responses. Between the two intranasal groups delivering MhCP, the IN:Spore+MhCP group exhibited a tendency to enhance the immune response earlier and maintained higher levels of PlpE- and NLKT-specific IgA until the end of the study. This suggests that the binding of MhCP to *Bacillus* spores led to an augmented immune response, emphasizing spores as effective adjuvants. Ayalew and colleagues (2009) conducted an animal trial where weaned beef calves were immunized intranasally, on days 0 and 14, with the R2-NLKT-R2-NLKT chimeric protein, mixed with and without native cholera toxin^35^. The same study also included another treatment group where calves were vaccinated intranasally with CTB appended chimeric protein, CTB-R2-NLKT^35^. Their study revealed that vaccination with these chimeric proteins enhanced resistance to intrabronchial challenge with *M. haemolytica* and induced antibody responses against the bacterium. However, significant increases in antibody responses against individual constructs were observed only following bacterial challenge^35^. Although our study did not involve challenging sheep with *M. haemolytica*, the significant increase in immune responses observed following booster immunization of sheep with intranasal Spore-MhCP features the effectiveness of the spore-based technology utilized in this study. A recent attempt has been made to develop a recombinant bovine herpes virus-1 (BHV-1) vectored vaccine expressing PlpE-LKT chimeric protein^37^. Although anti-LKT antibodies were produced following intranasal vaccination of bighorn sheep with the BHV-1 vectored vaccine, inconsistent development of antibodies against surface antigens and failure of the vaccine to protect against *M. haemolytica* challenge emphasize the importance of antigen delivery in immunization^38^. The notable enhancements in immune responses observed in the intranasal spore-bound group, following booster immunization in our study, highlight the effectiveness of the *B. subtilis* spores in augmenting immune responses as an adjuvant.

In-feed or oral vaccines hold significant appeal for the livestock industry due to their ease of administration, requiring minimal expertise and time to administer to livestock. Despite the limited effectiveness of intragastric delivery of Spore-MhCP in mice^23^, we explored its in-feed delivery in sheep. The rationale was that prolonged rumination in sheep could potentially allow antigens to remain in the upper digestive tract long enough to stimulate laryngeal lymphoid tissue^36^, which could result in immunity through the upper respiratory tract instead of the lower gastrointestinal tract. In this regard, we fed sheep a high-fiber alfalfa-based diet to promote prolonged rumination. Compared to the pre-immunization titers, IG:Spore+MhCP group resulted in significantly higher PlpE- and NLKT-specific IgA in BAL and fecal samples after receiving the third round of immunization, and remained elevated until the end of the study. The immune response generated from the in-feed immunization of Spore-MhCP vaccine is a noteworthy finding. This indicates that, when bound to spores, the recombinant protein resisted degradation sufficiently for both LktA and PlpE antigenic components to be taken up by M cells, activating immunocompetent cells in the mucosa-associated lymphoid tissue^39^. Additionally, the presence of antigen-specific secretory antibodies in the lungs of sheep from the IG:Spore+MhCP group suggests the possible trafficking of stimulated immune cells from the gut, while antigen-specific IgA in feces may indicates a local response^36^. While it was not possible to determine whether the immune activation of the IG:Spore+MhCP group occurred from laryngeal activation or trafficking from the gut, it could have been a combination of both. Unlike IN:Spore+MhCP group, in-feed group required three rounds of immunizations. One of the limitations of this study was the absence of an in-feed unbound antigen group. However, the strong increase in immune responses in the lungs after the third in-feed Spore+MhCP vaccination suggests that it may be effective in reducing the severity of lung infection by *M. haemolytica*. While vaccine tolerance can potentially occur through oral immunization, the IG:Spore+MhCP group had increased systemic and mucosal immune responses compared to pre-immunization levels. *B. subtilis* spore-based vaccines have been shown to elicit balanced Th1/Th2 immunity, which is important for optimal antibody production and mitigating oral tolerance^40^. Combined, the risk of oral tolerance therefore seems limited for IG:Spore+MhCP, at least to the time points evaluated in this study.

Nasopharyngeal microbiota profiling showed the most prevalent phyla identified in the nasopharynx of sheep were Proteobacteria, Bacteroidota, Firmicutes, and Actinobacteriota. This finding aligns with previous studies that have also observed Proteobacteria, Bacteroidota, and Firmicutes as predominant phyla in the nasopharynx^41^ and lungs^42^ of sheep. The predominance of *Mycoplasma*, *Moraxella*, and *Mannheimia* as the most abundant genera in our study is also consistent with findings from previous reports in sheep^41,42^. The composition and structure of the microbial communities in the nasopharynx exhibited changes over time, likely influenced by the vaccination process. The intranasal vaccination with Spore-MhCP altered 27 genera. Among them, 8 genera showed a decrease in abundance, while 7 showed an increase in abundance for more than a single time point, compared to the Control treatment. Most notable was a consistent decrease of *Mannheimia* in the IN:Spore+MhCP group compared to the Control group, which could be attributed to the heightened antibody levels induced by intranasal immunization with spore-MhCP. While it is difficult to explain, this spore-bound intranasal vaccine also demonstrated a reduction in the abundance of important pathogenic genera, including *Moraxella*, *Mycoplasma*, and *Staphylococcus*, at one or more of the observed time points. The probiotic *Bacillus* spores utilized in the Spore-MhCP vaccine formulation may potentially have a contributory effect. *Bacillus* is used as a gastrointestinal probiotic in both human and livestock, and is known for its potential health benefits^43^. Probiotics are generally believed to positively influence the microbiota in a way that promotes host well-being. While *Bacillus* has been used as a probiotic to decrease pathogens^44^, it did not increase in abundance after intranasal administration of Spore-MhCP and spores did not germinate in the sheep's upper respiratory tract. Therefore, it is likely that changes observed in microbiota were due to specific (i.e. *Mannheimia*) and general (innate) immune responses from host interaction with this Spore-MhCP vaccine. The spore-based technology presents an opportunity to integrate immunologically significant antigens from additional pneumonia or BRD-causing pathogens like *Pasteurella multocida* and *Histophilus somni*. This strategy holds potential for creating a comprehensive multivalent vaccine for ruminants, providing immunity against a wider range of respiratory diseases.

Intranasal vaccination with spore-bound antigen resulted in mucosal and systemic antigen-specific antibody production against *M. haemolytica*. Combined with the analysis of the sheep nasopharyngeal microbiota, secretory IgA to *M. haemolytica* resulting from IN:Spore+MhCP showed potential to reduce colonization by this pathogen. In addition, the IG:Spore+MhCP highlighted the potential of in-feed vaccines to mitigate ruminant respiratory pathogens. While further research is needed, large-scale administration of an oral vaccine would be particularly relevant to livestock management strategies limited by cost and ease-of-use. Since the proliferation of *M. haemolytica* in the upper respiratory tract is a prerequisite to lung infection, this spore-bound vaccine may offer protection against pathogen growth and subsequent infection. Future studies are warranted to fully evaluate the efficacy of this spore-based vaccine against *M. haemolytica*, where vaccinated cattle will be challenged with a primary viral pathogen, bovine herpesvirus-1, followed by upper respiratory tract inoculation with *M. haemolytica*^45^. This represents a stress-induced challenge model and will enable evaluation of vaccine effectiveness on pathogen colonization and infection in both the upper and lower respiratory tracts. Furthermore, while immune responses can be hindered by passive or pre-existing immunity^38^, this current study demonstrated that intranasal immunization of Spore-MhCP still elicited a strong immune response despite sheep having detectable levels of antibodies at the beginning of the study. This vaccine may therefore be relevant to cattle displaying passive immunity, such as newborn calves, or cattle that have been exposed naturally to *M. haemolytica*. While the premise was to use sheep as a ruminant model for cattle, the intranasal Spore-MhCP vaccine is also applicable to mitigating *M. haemolytica* respiratory infections in sheep. However, evaluation for cross-protection against ovine virulent serotypes (*M. haemolytica* serotype 1 and 2) would first be needed.

## Methods

### Vaccine (Spore-MhCP) preparation

The *M. haemolytica* chimeric protein MhCP (CTB-PlpE-NLKT-PlpE-NLKT), used in vaccine formulation, was comprised of a truncated cholera toxin B subunit (CTB) along with two copies each of the immunodominant surface epitope (R2) from *M. haemolytica* PlpE and the neutralizing epitope of leukotoxin (NLKT)^23^. MhCP had a calculated molecular weight of 56.4 kDa and a pI of 9.1. The chimeric protein MhCP was synthesized, purified and then adsorbed to *B. subtilis* spores, to use as vaccine (Spore-MhCP). The preparation of spores along with the construction, expression and purification of MhCP have been described previously^23^. The adsorption of the chimeric protein MhCP to *B. subtilis* spores was conducted following previously established optimized conditions^23^, where 100 µg of MhCP was adsorbed to 2×10^10^ spores in phosphate-buffered saline (PBS; pH 4). The resultant spore-bound vaccine Spore-MhCP was evaluated in sheep.

### Animals and experimental design

The work described in this study was reviewed and approved by the Lethbridge Research and Development Centre (LeRDC) Animal Care Committee (Animal Use Protocol # 1906) before commencement of the trial. This study was carried out in strict accordance with the recommendations established in the Canadian Council on Animal Care Guidelines^46^, and complied with the ARRIVE guidelines. Forty-eight male sheep, approximately 4 months old and of similar body weight (37.2 ± 1.9 kg), were sourced from the LeRDC herd, and housed in individual pens at the sheep barn. The sheep were randomly assigned to six experimental groups (*n* = 8 per treatment): intramuscular free MhCP (IM:MhCP; positive control to measure systemic immunity of MhCP in native form, without spores); intranasal spore-bound MhCP (IN:Spore+MhCP); intranasal free MhCP without spore (IN:MhCP); intranasal spore without any MhCP (IN:Spore); intragastric spore-bound MhCP delivered in feed (IG:Spore+MhCP); and control sheep (Control; no MhCP administered, negative control, received PBS intranasally). Details of the experimental design are listed in Table 1 and depicted in Supplementary Fig. 2.

**Table 1:** Experimental design in which sheep were immunized with *Mannheimia haemolytica* chimeric protein MhCP (CTB+PlpE+NLKT+PlpE+NLKT) via different routes, either in free form or bound to *B. subtilis* spores^a^

Treatment group	No. of Sheep	Route of administration	Vaccine formulations (amount per dose)	Samples collected
IM: MhCP	8	Intra-muscular	MhCP (100 µg) mixed with IFA; No Spore	Blood, BAL, NS, Feces
IN: Spore+MhCP	8	Intra-nasal	Spore (2 × 10^10^) bound MhCP (100 µg)	Blood, BAL, NS, Feces
IN: MhCP	8	Intra-nasal	MhCP (100 µg) only; No Spore	Blood, BAL, NS, Feces
IN: Spore	8	Intra-nasal	Spore (2 × 10^10^) only; No MhCP	Blood, BAL, NS, Feces
IG: Spore+MhCP	8	Intra-gastric/In-feed	Spore (2 × 10^10^) bound MhCP (100 µg)	Blood, BAL, NS, Feces
Control	8	Intra-nasal	PBS; No MhCP; No Spore	Blood, BAL, NS, Feces

^a^Sheep were immunized on days 0 and 14, with the exception of the Intra-gastric/In-feed group, which was immunized on days 1, 2, 14, 15, 28, and 29. A total of 100 µg antigen was administered per sheep per immunization time point. Blood, nasal secretions (NS), and fecal samples were collected on days 0 (prior to vaccination), 7, 14 (prior to vaccination), 21, 28, 35, and 42. Bronchoalveolar lavage (BAL) samples were also collected on days 0, 14, 28, and 42.

### Immunizations and sample collection

Sheep were immunized on days 0 and 14 with the exception of IG:Spore+MhCP (in-feed) group which was immunized on days 1, 2, 14, 15, 28, and 29. A total of 100 µg of antigen MhCP was administered per sheep per immunization time point. The IM:MhCP treatment group received antigen MhCP mixed with Incomplete Freund’s adjuvant (IFA) in a 1:1 ratio. For intranasal vaccinations, the treatments were delivered as a liquid spray, with spore-bound antigen, free antigen, and spores suspended in 2 ml of PBS, loaded into an LMA® MADgic® Laryngo-Tracheal Mucosal Atomization Device, and 1 mL was sprayed into each nasal cavity. After inoculation, the animal’s head was held upright for one minute to ensure that the treatment remained in place^47^. For IG:Spore+MhCP (in-feed) vaccination, the spore-bound antigen was suspended in PBS and sprayed onto 500 g of alfalfa pellet and offered to sheep prior to the standard diet, to ensure complete consumption. The Control group received 2 ml of PBS by intra-nasal spray, with 1 ml applied to each nasal cavity. Blood, deep nasal swab (left nostril), and fecal samples were collected on days 0 (prior to vaccination), 7, 14 (prior to vaccination), 21, 28, 35, and 42 from all treatment groups. Bronchoalveolar lavage (BAL) samples were also collected on day 0, 14, 28 and 42. To determine the immunogenicity of vaccination routes, samples were analyzed quantitatively for antigen-specific IgG and IgA. An additional deep nasal swab from the right nostril was collected from IN:Spore+MhCP and Control sheep on days 0, 7, 14, 21, 28, and 35 to evaluate the effects of the vaccine on the sheep respiratory bacterial microbiota. Given Canadian regulations concerning the use of unlicensed vaccines in livestock, all sheep were euthanized by a licensed veterinarian and disposed of at the end of the study. The sheep were first sedated with an intramuscular injection of xylazine (Rompun 20 mg/mL, Elanco; 0.50 mL/45 kg of body weight), and subsequently euthanized by intravenous administration of an overdose of pentobarbital (Euthanyl Forte 540 mg/mL, Bimeda MTC; 1 mL/5 kg of body weight) via the jugular vein. After euthanasia, all the carcasses were incinerated.

### Quantification of antigen-specific serum IgG

Antigen-specific IgG in sheep serum was quantified using enzyme-linked immunosorbent assay (ELISA). Individual components of the chimeric constructs (i.e., PlpE and NLKT) were synthesized commercially (BioBasic Inc., Markham, Canada). The sequences were optimized for expression in *Escherichia coli* and purified using immobilized metal affinity chromatography. The purified recombinant antigens, PlpE and NLKT, were suspended in coating buffer and used as ligands to coat ELISA plates at a concentration of 50 ng per well. The plates were then incubated overnight at 4°C. After washing the plates, blocking solution (50 mM Tris, 0.14 M NaCl, 0.05% Tween 20, pH 8.0) was added and incubated for an hour at room temperature. Serum samples were diluted in sample buffer (50 mM Tris, 0.14 M NaCl, 0.05% Tween 20) and 100 µL aliquots of each dilution were then added to wells in duplicate, followed by another hour-long incubation at room temperature. After the addition of horseradish peroxidase-conjugated secondary antibody (A50-104P-24; Bethyl Laboratories Inc.), the plates were incubated at room temperature for an hour. The color development was carried out using TMB (3,3´,5,5´-tetramethylbenzidine; ThermoFisher, Canada). The reactions were halted by adding ELISA stop solution (0.2 M H2SO4), and the optical densities of the reactions were measured at 450 nm using a Synergy HTX multi-detection microplate reader (BioTek Instruments Inc., Winooski, Vermont, USA) and analyzed with Gen5 software (BioTek Instruments Inc., Winooski, Vermont, USA). Concentrations were determined using a standard curve generated from a reference serum (RS10-108-2; Bethyl Laboratories Inc.).

### Quantification of antigen-specific secretory IgA

BAL and fecal samples were pre-processed to quantify IgA as previously described^48^. The nasal swabs were pre-processed by incubating in PBS containing 0.1% BSA, 0.05% Tween 20, and 1 mM phenylmethylsulphonyl fluoride (PMSF) at normal room temperature for 1 h, followed by centrifugation at 13,000 X g for 10 min. The resulting supernatant obtained from these processes was utilized to measure mucosal secretory IgA (sIgA) concentrations by ELISA. For the determination of IgA, 96-well ELISA plates were coated with 50 ng/well of purified recombinant antigens NLKT and PlpE separately, and incubated at 4 °C overnight. After a blocking solution was added, as described for IgG, samples were pipetted into individual wells in duplicate, followed by incubation at room temperature for 1 h. The plates were then washed, and an HRP-conjugated secondary antibody (AHP949P; BioRad, Canada) was added, followed by an additional incubation period of 1 h. TMB substrate was added, and after color development, the reaction was stopped by the addition of a stop solution. The plates were read at 450 nm using a Synergy HTX multi-detection microplate reader (BioTek Instruments Inc, Winooski, Vermont, USA) to determine optical density. Concentrations were determined using a standard curve generated from a sheep IgA standard (CLFA06, Cedarlane, Canada).

The ELISA data were analyzed using SAS PROC GLIMMIX (SAS version 9.4, SAS Inst. Inc., Cary, NC). For each generalized linear model, a distribution from the exponential family (gamma, inverse Gaussian, log-normal, normal, exponential, or shifted *t*) was selected based on the respective values of the Bayesian information criterion (BIC). A lower value of the BIC associated with the model indicated superior model fit. Statistical significance was indicated by *p*-values less than 0.05. Microsoft Excel 2010 and GraphPad Prism version 10.1.0 were utilized for the visual representation of all ELISA analyses.

### DNA extraction, amplicon sequencing, and analysis

Nucleic acids were extracted from nasopharyngeal (NP) swabs using the method previously described^49^. DNA was amplified using primers 515-F (5′-GTGYCAGCMGCCGCGGTAA-′3) and 806-R (5′-GGACTACNVGGGTWTCTAAT-′3) targeting the V4 region of the 16S rRNA gene, as described earlier^50^. The amplicon was sequenced on a MiSeq instrument (PE250) using the MiSeq Reagent Kit v2 (2 ×250 bp; Genome Quebec, Montreal, Quebec, Canada). The expected library size was 25,000 reads per sample.

Data quality assessment of the unprocessed reads was executed using FastQC v0.11.9 and MultiQC v1.12^51^. Sequence trimming was conducted using Trimmomatic v0.39^52^. Trimmomatic eliminated primer sequences and low-quality reads, employing the following parameters: HEADCROP:10, SLIDINGWINDOW:5:20, LEADING:20, and TRAILING:20. Subsequent statistical analyses were carried out using R version 4.2.2^53^. Following sequence trimming, the paired reads underwent additional filtering utilizing the DADA2 v1.22.0 ‘filterAndTrim’ function with default parameters and subsequently merged. Bimeric sequences were excised from the merged dataset using the DADA2 ‘removeBimeraDenovo’ function. Taxonomic classification of the 16S sequences was achieved utilizing DADA2’s ‘IdTaxa’ function and the SILVA 138 database^54^, which created the amplicon sequence variants (ASVs) table. Data manipulation was conducted using Phyloseq v1.42.0^55^, and figures were generated with ggplot2 v3.4.1^56^. The α-diversities were computed via ‘vegan’ v2.6-4^57^ in the form of the Shannon diversity index and richness (Chao1), and corresponding plots were generated. To reduce noise in downstream analysis, the ASV table was filtered to retain ASVs present with a count of at least 2 in a minimum of 1% of the samples. One-way analysis of variance (ANOVA) was employed to assess α-diversity with respect to treatment and time. The package ‘vegan’ was used to calculate β-dispersion and permutational multivariate analysis of variance (PERMANOVA) with 9999 permutations to examine the effects of treatment at each sampling time on the structure of the microbial community. β-diversity was calculated from the filtered ASV counts after they were subjected to size factor normalization using ‘GMPR’ within DESeq2 v1.40.2^58^. Sample-to-sample distances were determined with the Bray-Curtis metric through Phyloseq ordination and plotted as a detrended correspondence analysis (DCA). Significant [*p* < 0.01] differentially abundant genera were identified with DESeq2 by fitting a negative binomial model to the equation sampling time X vs. Day 0 with respect to treatments IN:Spore+MhCP vs Control (“~Treatment + Time + Time:Treatment”), and visualized with heatmaps.

## Supplementary information

Supplemental Figures

## Data Availability

The raw sequencing data used in this article have been submitted to the NCBI Sequence Read Archive (SRA) under the BioProject accession ID PRJNA1194181 (http://www.ncbi.nlm.nih.gov/sra).
