# Supplementary material for: Effect of a spore-based Mannheimia haemolytica vaccine on immune responses and respiratory microbiota in sheep
Source: NPJ Vaccines. 2026 Jul 10;11:144. doi: 10.1038/s41541-026-01415-x (PMC13354553; doi:10.1038/s41541-026-01415-x)
Supplement: Supplementary file 1 — Supplemental Figures [file 41541_2026_1415_MOESM1_ESM.pdf]

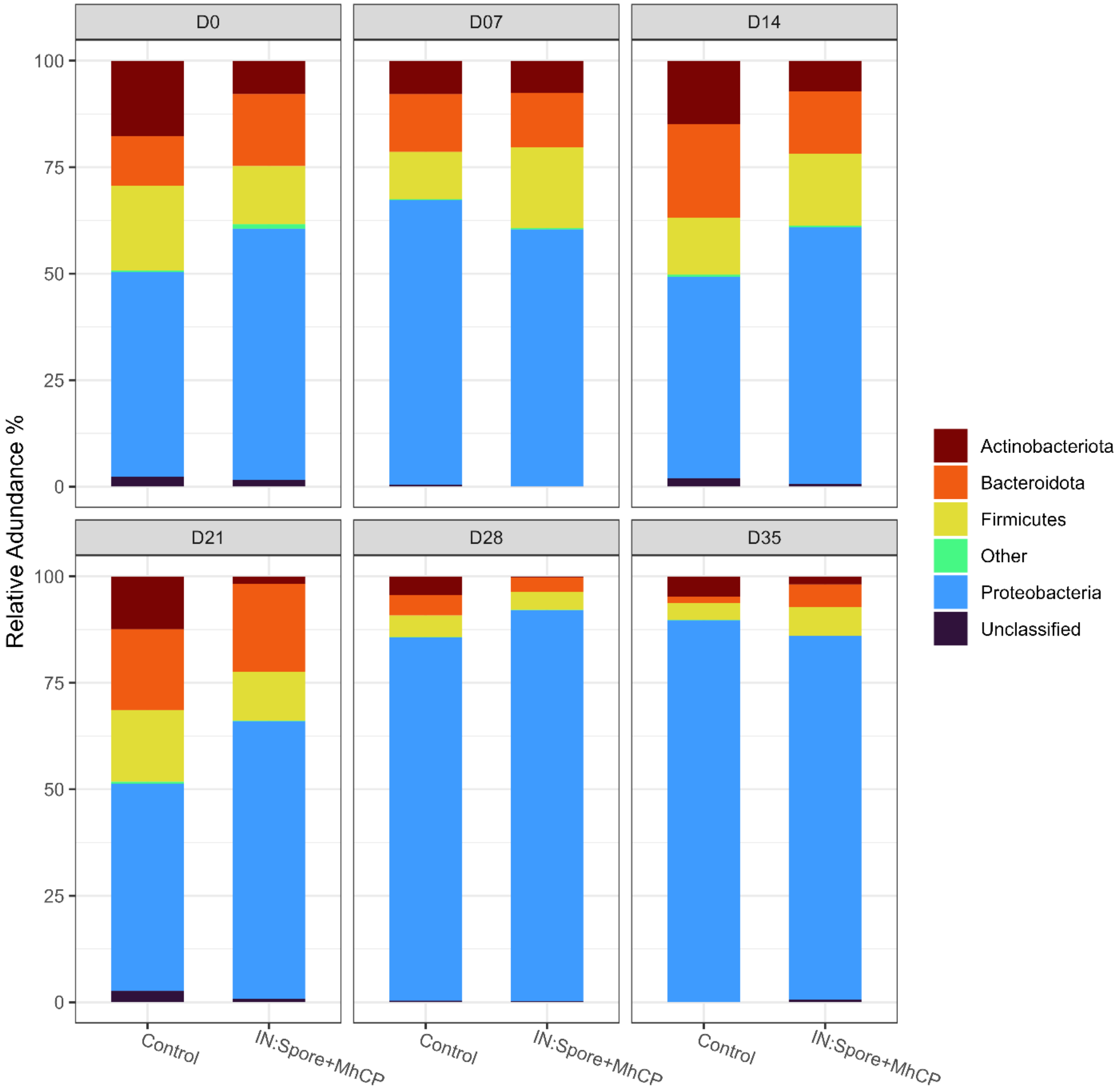

Supplemental Figure 1. Relative abundance of the phyla in nasopharyngeal samples from vaccinated sheep. Sheep were immunized on days 0 and 14. Deep nasal swabs (from right nostril) were collected from Intranasal spore-bound antigen (IN:Spore+MhCP) and Control/ Naïve (Control; administered intranasal saline) treatment groups on days 0, 7, 14, 21, 28, and 35 and analyzed by sequencing of the 16S rRNA gene.

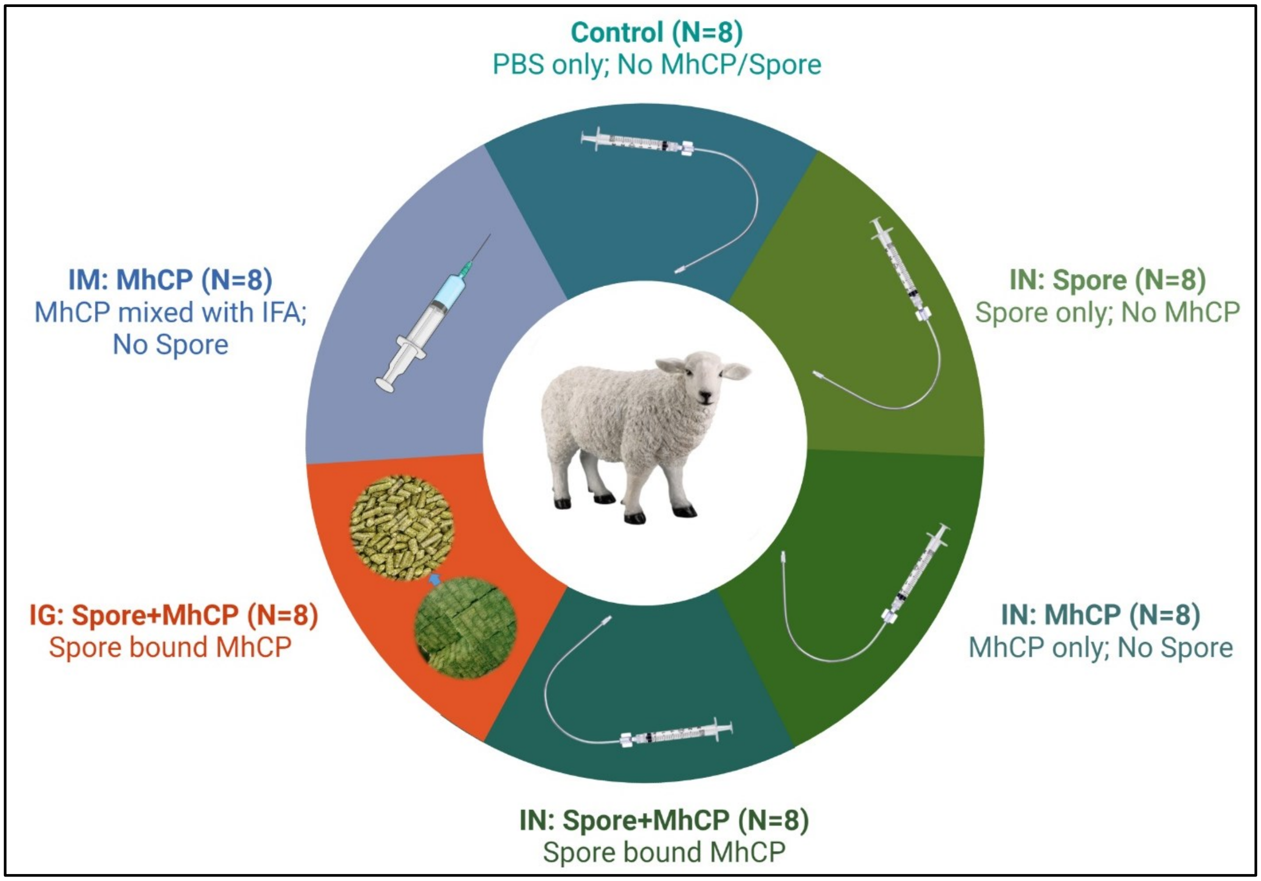

Supplemental Figure 2. Experimental design and the immunization methods. Sheep were immunized on days 0 and 14, except the IG:Spore+MhCP (in-feed) group, which was immunized on days 1, 2, 14, 15, 28, and 29. Each immunization included 100 µg of MhCP antigen per sheep. The IM:MhCP group received MhCP mixed with Incomplete Freund's Adjuvant (IFA) in a 1:1 ratio. Intranasal immunizations were administered as a liquid spray using an atomization device, with 1 mL delivered per nasal cavity. For the IG:Spore+MhCP (in-feed) group, the spore-bound antigen was sprayed onto 500 g of alfalfa pellets and fed before the standard diet. Control group received 2 mL of PBS via intranasal spray. This figure is created in BioRender (https://BioRender.com/l70k670).
